# Editorial: Strengthening food labeling policies in Brazil

**DOI:** 10.3389/fnut.2023.1196243

**Published:** 2023-05-04

**Authors:** Fabio da Silva Gomes, Camila Corvalán, Rosires Deliza, Simon Barquera

**Affiliations:** ^1^Pan American Health Organization/World Health Organization, Department of Noncommunicable Diseases and Mental Health, Washington, DC, United States; ^2^Center for Food Environment Research (CIAPEC), Institute of Nutrition and Food Technology (INTA), University of Chile, Santiago, Chile; ^3^Embrapa Agroindústria de Alimentos, Rio de Janeiro, RJ, Brazil; ^4^Centro de Investigación en Nutrición y Salud, Instituto Nacional de Salud Pública (INSP), Cuernavaca, Mexico

**Keywords:** food labeling, food and nutrition policies and programs, food legislation, public health, healthy diets, malnutrition

Food labeling policies can have different purposes, but consumers' right to protection, human rights to health and to adequate food, the rights of the child and all other interdependent human rights prevail. For this reason, research that seeks to inform the best policy options in fulfilling, protecting, and respecting these rights is paramount.

Brazil is known for novel research and action that have led to great advance of knowledge and policies on nutrition and health globally. Different sorts of edible and drinkable products have been historically defined as foods and manufactured and labeled to mimic foods.

Unhealthy edible and drinkable commodities industry have insisted on the use of market-forged categorization of products with the purpose of demonstrating an artificial diversification of alike products, for which labeling has been an instrument of consumer deception.

Labeling is often used to distort the real composition of products. Batti et al. found that half of the food products they assessed in Brazil that highlighted the term whole grain or related expressions on the front label did not have a whole-grain ingredient listed in the first position of the ingredients list. Barros et al. also revealed how the ingredients are listed on labels in ways that hide products real content and composition, showing that the use non-specific terms for listing industrially produced trans fatty acids (i-TFA) ingredients in foods that are sources of i-TFA increased in Brazil. Prates et al. have shown how the use of nutrition claims convey such deception, corroborating previous findings. Unfortunately, the labeling regulation in Brazil still allows the use of claims in products that are not recommended as part of a healthy diet, as Mais, Borges et al. described. Along these lines, Sato et al. also highlighted in their paper the importance of regulating other persuasive elements that can strengthen deception, such as mascots and cartoon characters.

Another important component of food products labeling not yet regulated in Brazil and most countries worldwide is branding. Alcantara et al.'s findings show brands should also be subject to regulation to help consumers take a more critical and better informed decision.

Mais, Borges et al. and Coutinho et al. also described others regulatory gaps in the Brazilian regulation, which resulted from interference of opposing commercial actors. Mais, Mialon et al. described the tactics and arguments of these opponents, which were mostly centered on a misconception of freedom of choice, used to favor regulated actors' interests.

Front-of-package labeling can be a powerful policy tool to improve food choices, but the ultimate outcome on how it can effectively improve diets and public help, depends on the system used and the criteria to define the products subject to regulations. This Research Topic highlights how Brazilian regulatory authorities failed to adopt the best policy option. Borges et al. and Tomaz et al. have shown Brazil has adopted one of the most permissive criteria to define products subject to regulation in Latin America, allowing three times more ultra-processed food products to omit warnings for sodium, four times more sugar-sweetened beverages and six times more dairy drinks to omit warnings for sugar, compared to the gold standard for the Region of the Americas (i.e., the Nutrient Profile Model of the Pan-American Health Organization) (1), which has been adopted by Mexico and other countries in the Region.

Silva et al. and other research have been extrapolating findings from the virtual environment and e-commerce to physical environments, and results tend to indicate that front-of-package labeling originally designed for to be applied to real size products, may need adaptations when used in virtual environments to preserve their effectiveness.

Santana et al.'s findings help us understand how warnings on excessive amounts of sugar help populations avoiding products with such warnings as they recognize the harmful effects of sugar intake. In assessing the best option to deliver this information to consumers and make it timely and relevant to their decision, the octagonal warning label performs best according to Scapin et al.. Fernandes et al. confirmed that, by demonstrating with objective measures from electroencephalograms, that textual warnings have the ability to neutralize strongly positive emotions triggered by ultra-processed products, allowing consumers to make a more critical appraisal of products.

The implications of the research featured in this topic, also highlights the importance of recognizing Brazil's work and focus on supporting, promoting and protecting healthy and adequate eating. The NOVA food classification (2) and the Brazilian dietary guidelines (3, 4) were the first to draw the world's attention for the need to bring a new purpose to how foods are categorized. So that policies can drive support, promote, and protect diets that are based on foods and the culinary preparations enabled by the great sociobiodiversity found in Brazil and worldwide.

This implies the importance of regulating ultra-processed products, by means of effective labeling and other policies that can have their application facilitated by such labeling regulations. An effective front-of-package warning label can help reducing demand for ultra-processed products, but can also ease the application, monitoring and enforcement of marketing and school food policies aimed at reducing demand for and offer of products featuring such warnings.

In addition, Brazil's official nutrition recommendations also call the attention for preventing distortion of policies that could mislead to an impression that policies that are actually designed to reduce demand and offer of ultra-processed products are aimed at reformulating them. Such distortion could deviate the national policy from its main mandate, jeopardizing the support, promotion, and protection of healthy diets based on unprocessed and minimally processed foods and culinary preparations and not on ultra-processed products being them reformulated or not.

## Author contributions

FG wrote the first draft of the manuscript. All authors contributed to the conception of the editorial, manuscript revision, read, and approved the submitted version.
